# Oral Chronic Graft-Versus-Host Disease: Pathogenesis, Diagnosis, Current Treatment, and Emerging Therapies

**DOI:** 10.3390/ijms251910411

**Published:** 2024-09-27

**Authors:** Joe T. Nguyen, Maryam Jessri, Ana C. Costa-da-Silva, Rubina Sharma, Jacqueline W. Mays, Nathaniel S. Treister

**Affiliations:** 1 Nguyen Laboratory, Head and Neck Cancer Section, Surgical Oncology Program, Center for Cancer Research, National Cancer Institute, National Institutes of Health, Bethesda, MD 20892, USA; 2 Oral Immunobiology Unit, National Institute of Dental and Craniofacial Research, National Institutes of Health, Bethesda, MD 20892, USA; ana.costadasilva@nih.gov (A.C.C.-d.-S.); rubina.sharma@nih.gov (R.S.); jacqueline.mays@nih.gov (J.W.M.); 3 Metro North Hospital and Health Service, Queensland Health, Brisbane, QLD 4029, Australia; maryam.jessri@health.qld.gov.au; 4 Department of Oral Medicine and Pathology, School of Dentistry, The University of Queensland, Herston, QLD 4072, Australia; 5 Division of Oral Medicine and Dentistry, Brigham and Women’s Hospital, Boston, MA 02115, USA; 6 Department of Oral Medicine, Infection and Immunity, Harvard School of Dental Medicine, Boston, MA 02114, USA

**Keywords:** cGVHD, chronic graft-versus-host-disease, oral chronic graft-versus-host-disease, oral cGVHD, ibrutinib, ruxolitinib, belumosudil

## Abstract

Chronic graft-versus-host disease (cGvHD) is a multisystem disorder that occurs in recipients of allogeneic hematopoietic (alloHCT) stem cell transplants and is characterized by both inflammatory and fibrotic manifestations. It begins with the recognition of host tissues by the non-self (allogeneic) graft and progresses to tissue inflammation, organ dysfunction and fibrosis throughout the body. Oral cavity manifestations of cGVHD include mucosal features, salivary gland dysfunction and fibrosis. This review synthesizes current knowledge on the pathogenesis, diagnosis and management of oral cGVHD, with a focus on emerging trends and novel therapeutics. Data from various clinical studies and expert consensus are integrated to provide a comprehensive overview.

## 1. Introduction

**Figure 1:**
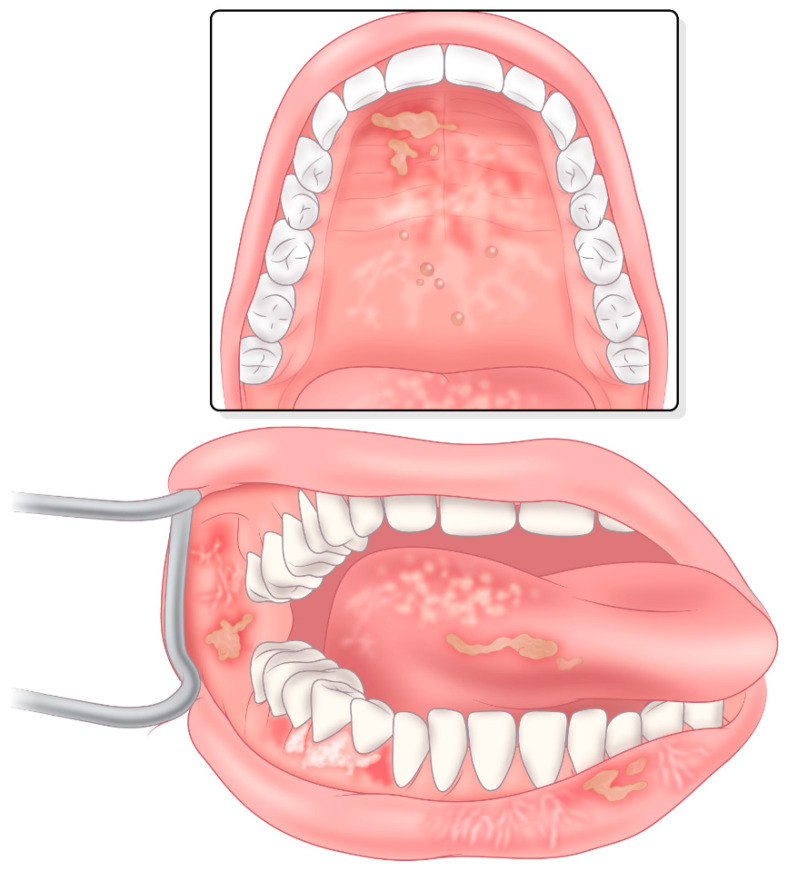
Clinical features of oral mucosal chronic graft-versus-host disease. Typical features of oral mucosa chronic GVHD include lichen planus-like alterations typified by the presence of white reticular streaks or lace-like lines, erythema and ulcerations illustrated here on the buccal mucosa, lower lip, lateral tongue and palate. Superficial transient mucoceles may be present on the palate and lower labial mucosa.

Chronic graft-versus-host disease (cGVHD) affects 30–70% of survivors of allogeneic hematopoietic stem cell transplantation (alloHCT) [1,2,3,4,5]. cGVHD is an alloreactive disease that begins with recognition of host tissues by the non-self (allogeneic) graft and progresses to tissue inflammation and fibrosis. Manifestations of cGVHD are present in the oral cavity in 45–85% of cGVHD patients and include oral mucosal features, salivary gland dysfunction and fibrosis (Figure 1) [6]. Significant heterogeneity exists among patients with cGVHD [7], including in their underlying indication for transplant, the therapeutic protocols used, their disease phenotypes and potentially in the underlying pathogenesis [8,9]. This review provides an in-depth overview of oral cGVHD pathogenesis, diagnosis and management and considers the potential impact of emerging trends and novel therapeutics.

## 2. Pathogenesis of Oral cGVHD

The pathogenesis of cGVHD involves multiple dysregulated biological processes and immune responses, fundamentally characterized by an alloreaction between the graft donor cells and host tissues. A three-phase model of cGVHD pathogenesis starts with conditioning-mediated tissue damage, followed by chronic inflammation and dysregulated immunity caused by loss of central and peripheral tolerance, progressing to fibrosis with impaired wound healing of the affected tissues [10]. Heterogeneity in pathophysiology translates to a pleomorphic and multifaceted disease; however, how and why the oral cavity, versus other target organs, is variably affected, remains unknown.

### 2.1. Initial Events

Prior to alloHCT, conditioning regimens, which may include chemotherapy, total body irradiation and other agents, can prime the immune system for early events of GVHD [11]. These regimens result in tissue damage that generates pathogen-associated molecular patterns (PAMPs) as well as damage-associated molecular patterns (DAMPs); these may serve as GVHD-triggering events [12,13]. Pattern recognition receptors (PRRs) on innate immune cells recognize DAMPs and PAMPs, leading to immune system activation [14,15]. In mouse models, infusion of anti-inflammatory macrophages (trained using low-level exposure to a DAMP (i.e., toll-like receptor 4) agonist) reduced GVHD, while pro-inflammatory macrophages (exposed to Bacillus Calmette–Guérin components) worsened GVHD [14]. These experiments illustrate how previous damage and inflammation such as acute GVHD (aGVHD), exposure to cytotoxic therapies and infections could be associated with an elevated risk of cGVHD.

Antigen-presenting cells (APCs) play an important role in activating, co-stimulating and expanding alloreactive T cells through molecule recognition (including PAMPs) and pro-inflammatory cytokine release (e.g., tumor necrosis factor (TNF), Interleukin (IL-1) and IL-6) [16,17,18]. Primed APCs upregulate CD80/86 and provide co-stimulatory signals for full T-cell activation. APCs have been associated with GVHD onset in target tissues, including an increased frequency of activated monocytes/macrophages [19,20]. In preclinical mouse models for sclerodermatous cGVHD, defective antigen presentation by donor-derived conventional dendritic cells (DCs) contributed to impaired regulatory T cell (Treg) development, resulting in a loss of tolerance and promoting development of cGVHD [21]. Similarly, the alloreactive T-cell response impairs maturation of tolerogenic plasmacytoid DCs, which contributes to cGVHD development [22,23,24]. Finally, in cGVHD, foundational experiments showed that inhibiting Bruton’s tyrosine kinase (BTK) and Syk signaling in B cells is effective because B cells may incorrectly recognize antigens, which alters B-cell homeostasis and constitutively activates B cells [25,26].

### 2.2. Loss of Tolerance Leads to Dysregulated Immunity

Central and peripheral immune tolerance mechanisms are disrupted in cGVHD, permitting the pathogenesis and persistence of alloreactive immune cells. Thymic epithelial damage secondary to the conditioning regimen and/or aGVHD is an important step in the development of cGVHD, with subsequent impairment of negative selection of autoreactive cells and generation of thymic regulatory T cells [27,28,29,30,31,32]. Impaired central tolerance generates host-reactive T cells that, in addition to alloreactive cells present in the initial graft, further propagate tissue injury. In the case of de novo cGVHD (without preceding aGVHD), the initial inflammatory events may have been counterbalanced by tissue tolerance mechanisms [33] until unknown signals break this tolerance threshold and allow progression of alloreactivity. An imbalanced ratio between regulatory and effector T cells in peripheral blood [34,35,36] and tissue [37] is associated with development of cGVHD [38,39,40]. Preclinical and clinical studies demonstrate effective management using low-dose IL-2 therapy to expand Tregs [35,41,42,43].

### 2.3. Inflammation and Tissue Damage/Dysfunction

Chronic inflammation is a hallmark of cGVHD disease progression and contributes to tissue damage and dysfunction [44]. This inflammation is sustained by APCs that promote the inflammatory milieu via cytotoxic T-cell activation which, in turn, perpetuates tissue damage. Diverse subsets of CD4^+^ and CD8^+^ T cells have important roles in the initiation, propagation and tissue damage of cGVHD [45]. Donor T cells can differentiate into interferon gamma (IFNγ)- or IL-17-producing cells, generating Th1/Tc1 and Th17/Tc17 responses, respectively, that have been identified in elevated numbers in peripheral blood and target tissues of patients and in murine models of cGVHD [35,46,47,48,49,50,51,52]. Increased IFNγ expression, as well as downstream molecules of type I interferon signaling pathways, are reported in serum and tissues of cGVHD patients [53,54,55,56]. Preclinical models illustrate mechanisms of how IL-17 may contribute to skin [35,48,57,58], liver [59,60] and lung [61] GVHD, but the role of IL-17 is debated in some organs like the gut, where Th17 cells also play an important role in homeostasis [47,62]. In most cases, a mixed Th1/Th17 response is observed and improvement in cGVHD symptoms can be measured following blockade of IL-17 and IFNγ pathways using neutralizing mAbs [58,61,63], pharmacological inhibition [25,49,61,64,65,66,67] and knockout animals [49,61,68].

### 2.4. Fibrosis

Fibrosis is considered an end-stage phenomenon, characterized by an abnormal wound healing response to injury [69] and excessive deposition of extracellular matrix components, which leads to excess scar tissue formation, fibrosis and functional impairment [70]. The development of fibrosis is driven by chronic inflammation and immune dysregulation [71], where key cellular players, such as macrophages, myofibroblasts and mesenchymal stromal cells, are recruited and activated. In murine models of sclerotic cGVHD, macrophages recruited via CSF-1 and IL-17 assume a profibrotic phenotype [19,61,72] and induce mesenchymal stromal cell differentiation to fibroblasts using IL-1b and transforming growth factor-beta (TGF-β) [73]. In patients, pro-inflammatory cytokines and growth factors, including TGF-β [74] and IL-17 [75,76], play pivotal roles in this process by promoting the differentiation of fibroblasts into myofibroblasts. These myofibroblasts are responsible for the production of collagen and other matrix proteins, which accumulate in the oral tissues [77]. Following interaction between immune cells and fibroblasts, the fibrotic response is perpetuated as macrophages assume a profibrotic phenotype and enhance myofibroblast activity [78]. Direct evidence linking other cells to fibrosis, including T follicular helper cells and B cells, is lacking, although such involvement is suggested [79,80]. The resulting fibrosis not only disrupts the normal architecture of the oral mucosa but also leads to significant clinical manifestations, such as mucosal stiffness, restricted mouth opening and impaired oral functions, including speech and swallowing [81].

### 2.5. Oral cGVHD—Evidence from the Literature

**Figure 2:**
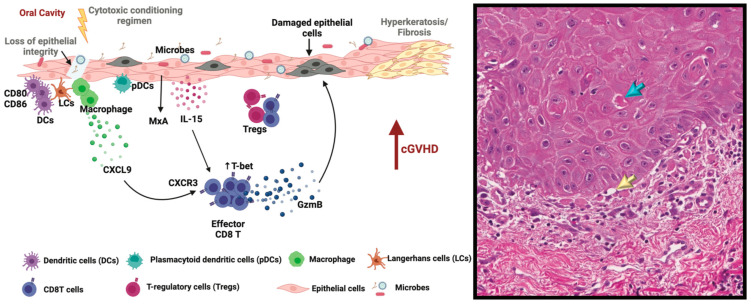
Oral mucosal cGVHD pathophysiology. Damage from the conditioning regimen, infection, dysbiosis, or injury prime the oral mucosa for alloimmune targeting via APCs. Primed APCs upregulate CD80/86 and provide co-stimulatory signals for T-cell activation. In parallel, macrophages upregulate the CXCL9/CXCR3 axis which regulates immune cell migration, differentiation and activation in the oral mucosa. Epithelial cells and other infiltrating cells secrete MxA and IL-15 which further support the expansion of effector T cells and direct alloimmune damage to the oral mucosa at the junctional epithelium (yellow arrow). Mucosal cGVHD effector CD8^+^ T cells have elevated T-bet (*T-box* expressed in T cells, *Tbx21*) expression and produce cytolytic granzyme B (GzmB) and are often found in proximity to damaged (blue arrow) and apoptotic epithelial cells. Epithelial hyperkeratosis and mucosal fibrosis may be present. Image referenced (magnification 20×) [91].

**Figure 3:**
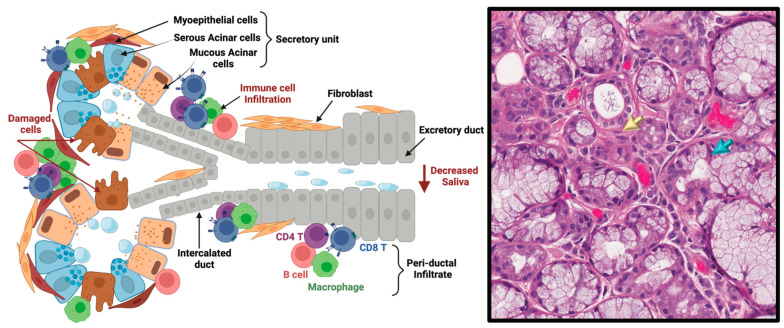
Salivary gland cGVHD pathophysiology. Infiltration of immune cells around the excretory ducts, which carry saliva from acinar units to the oral cavity, (yellow arrow) is a hallmark of oral cGVHD. Direct damage to acinar cells (blue arrow) and secretory units from immune effector cells, along with stromal infiltration of immune cells, is frequently observed in salivary gland cGVHD. Image referenced (magnification 20×) [91].

The oral mucosa and salivary glands are effector targets of cGVHD [82]. Oral mucosa cGVHD pathogenesis begins at the interface between the epithelium and connective tissue (Figure 2). At diagnosis, the most frequent histologic findings in oral cGVHD include degeneration of the basal layer of the epithelium, apoptotic bodies, lymphocytic infiltration and focal or total cleavage between the epithelial and connective tissue [83]. Salivary gland cGVHD is marked by infiltration or cuffing of immune cells around the excretory ducts, damage to the acinar cells and fibrosis (Figure 3) [84]. In the oral mucosa and salivary glands, cGVHD severity is associated with increased inflammatory cell infiltration, with a predominance of CD8^+^ T cells and monocytic (CD68) cells, but rare B cells [39,83,85,86,87,88,89,90].

As in the rest of the body, it is understood that damage from the conditioning regimen, infection, dysbiosis, or injury prime the oral mucosa and salivary glands for alloimmune targeting through APC activity (Figure 2). The presence of primed APCs, CD1a^+^ Langerhans cells [86,89,92,93] and plasmacytoid DCs [90] is detected in oral tissues, the latter being associated with type I interferon (IFN) signaling, including elevated expression of IL-15 and MIG and activation of STAT1 by keratinocytes in oral mucosa [90]. In parallel, macrophages upregulate the CXCL9/CXCR3 axis which regulates CD8^+^ T-cell migration, differentiation and activation in the oral mucosa. Epithelial cells and other infiltrating cells secrete MxA, a downstream product of the type I IFN signaling pathway and IL-15, which further support the expansion of effector T cells and direct alloimmune damage to the oral mucosa at the junctional epithelium. In cGVHD oral mucosa, effector CD8^+^T cells have elevated T-bet (*T-box* expressed in T cells, *Tbx21*) expression, produce cytolytic granzyme B (GzmB) and are often found in proximity to damaged and apoptotic epithelial cells. In oral mucosa and salivary glands, cGVHD clinical severity is associated with increased inflammatory cell infiltration, with a predominance of CD8^+^ T cells and monocytic (CD68) cells, but rare B cells [39,83,85,86,87,88,89,90]. Similarly, cGVHD oral mucosa contains T-bet^+^ type 1 effector T cells expressing CXCR3 [90] and, an increased number of FoxP3^+^ Treg cells expressing T-bet, CXCR3 and functional markers, such as ICOS and CD39, has been reported in oral mucosal tissues, despite comparable frequencies in peripheral blood between cGVHD patients and controls [39].

In cGVHD patients, activated B cells with increased survival capacity are present and signal through multiple pathways [94]. The anti-CD20 monoclonal antibody, rituximab, which depletes B cells, has reduced the severity of oral cGVHD symptoms in some patients [95]. An improvement in oral symptoms has been associated with increased numbers of peripheral IL-10-producing CD5^+^ B cells [96].

Saliva analysis demonstrates an elevation of pro-inflammatory cytokines, including a strong type I IFN signature and reduction of proteins from acinar cells in oral cGVHD [85,90,97]. In a proteomic study of saliva and salivary glands, a biomarker profile corresponding with structural damage to the salivary glands was identified at the onset of oral cGVHD [85]. Subsequent protein and molecular studies confirmed a significant reduction in zymogen granule 16B, ZG16B, in cGVHD saliva and salivary gland secretory cells [85]. However, little is known about the proteins found in saliva, suggesting future work is needed to understand the relevance or impact of these biomarkers.

### 2.6. Microbiome in Oral cGVHD

The role of the microbiome in the onset and persistence of oral cGVHD is an area of active investigation [98]. The oral and intestinal microbiomes undergo dysbiosis after alloHCT [99]. Studies correlating oral dysbiosis with cGVHD remain scarce. In a longitudinal pilot study, transient dysbiosis of the oral microbiome after alloHCT was related to oral mucositis but not oral cGVHD [100]. A recent multi-center study identified the expansion of *Streptococcus salivarius* and *Veillonella parvula* in the oral microbiome at the onset of oral cGVHD [101]. *Methylobacterium* species were enriched with severe oral mucositis and associated with oral dysbiosis in a microbiome/metabolomic assessment of 184 HCT patients [102]. In the setting of the gastrointestinal tract, more comprehensive work has associated gut microbiome perturbations with aGVHD incidence and has established an impact of antibiotic use and fecal transplantation on clinical aGVHD [103,104].

## 3. Diagnosis and Management of Oral Graft-Versus-Host Disease

### 3.1. Oral Mucosal cGVHD

The mucosal manifestations of oral cGVHD include lichen planus-like alterations typified by the presence of white reticular streaks or lace-like lines, erythema and ulcerations [105]. While any oral mucosal sites may be affected, those most frequently involved include the buccal mucosa, tongue and lips. While plaque-like features may be observed, including frank oral leukoplakia, it is uncertain if these lesions are at increased risk for malignant transformation [106,107]. Inflammatory changes can lead to the restriction of minor salivary gland ductal orifices, giving rise to superficial mucoceles which manifest as transient, saliva-filled blisters that typically resolve within hours. Symptoms may include increased sensitivity (to acidic, spicy, hard and crunchy food), pain (at rest and with speaking, mastication and when performing oral hygiene) and alteration and loss of taste. Furthermore, oral cGVHD can result in altered diet, an inability to maintain good oral hygiene and hence, an increased risk of dental and gingival pathology.

### 3.2. cGVHD Salivary Gland Dysfunction in Oral cGVHD

Salivary gland involvement is common and clinically distinct from oral mucosal disease [91,108]. In a study of 101 cGVHD patients, 77% reported xerostomia and 27% had salivary flow rates ≤ 0.2 mL/min 10^8^. Salivary gland dysfunction leads to lower salivary flow rates and dry mouth and is correlated with lower oral health-related quality of life, increased caries risk, impaired nutritional status, higher likelihood of oral candidiasis and decreased body mass index [109].

### 3.3. Fibrotic Oral cGVHD

This uncommon oral complication is characterized by a limited range of motion (tongue mobility and mouth opening) [110], mucosal fibrosis (fibrous bands and diffuse fibrosis), pain and secondary ulceration [6,111,112]. These features can negatively impact nutritional status, the maintaining and providing of oral hygiene and quality of life [113,114]. In a retrospective analysis of 39 patients with orofacial sclerodermatous cGVHD, all cases were preceded by lichenoid changes and 94% had a history of oral ulcerations [6], suggesting an important clinical link with longstanding chronic inflammation and development of tissue fibrosis. 

### 3.4. Diagnosis of Oral cGVHD

Oral cGVHD is a multifaceted disorder with clinical features resembling lichen planus, Sjögren syndrome and scleroderma; these may present in isolation or all at the same time [115]. According to the 2014 NIH Consensus Criteria, the oral mucosal lichen planus-like changes characterized by white lines and lacy-appearing lesions are considered sufficient to make a systemic diagnosis of cGVHD [105,115].

When diagnostic features are not prominent, a biopsy of the oral mucosa may be helpful to establish the diagnosis [105]. Minimal histologic [116] changes that support an oral cGVHD diagnosis include an accumulation of a band-like lymphocytic infiltrate at the epithelium–connective tissue interface, exocytosis, degeneration and apoptosis of the basal cells with colloid body formation [117]. The 2014 NIH Consensus Project refined the minimum pathognomonic histological criteria for oral mucosal cGVHD [84,117]. Nondiagnostic histopathologic features in cGVHD oral mucosal biopsies have been reported, such as flattening of rete ridges, intraepithelial inflammation, acanthosis, epithelial necrosis following vacuolization, apoptosis without colloid body formation, clefting or thickening of the basal membrane and increased keratinization [105,116,118]. Minor salivary gland histopathological features include intralobular or periductal lymphocytic inflammation and exocytosis of lymphocytes into intralobular ducts and acini. In addition, periductal fibrosis without generalized interstitial fibrosis is often present and acinar glands may be disrupted or fibrotic [117].

### 3.5. Clinical Scoring of Oral cGVHD

The Schubert Oral Mucosa Rating Scale was recommended by the National Institutes of Health (NIH) to assess and score disease severity in the oral cavity [119]. The 2005 NIH consensus included four manifestations of oral cGVHD, namely mucosal erythema (presence and severity), lichen planus-like changes (presence), ulcerations (percent of the affected mucosa) and mucoceles (total numbers) in its proposed oral mucosal score ^115^. Given that mucoceles were not reliably enumerated and their presence did not correlate with meaningful clinical outcomes, in 2014, the NIH Response Group recommended their removal from the oral mucosal score, leaving the revised 12-point objective clinical scoring system which accounts for (1) severity and extent of erythema, (2) extent of lichenoid hyperkeratotic changes and (3) extent of ulcerations [120].

The NIH Global Severity Score is a tool that grades eight different organs on a scale from 0 to 3 to evaluate the functional impact of cGVHD, with higher scores indicating greater disability [105]. An oral score of 1 indicates disease that is not significantly affecting nutritional intake, while a score of 3 reflects severe dietary restrictions due to oral symptoms [105]. Peak sensitivity to normally tolerated food or stimuli over the past week on a scale of 0 to 10, either alone or combined with the Lee cGVHD symptom scale [121,122], provides a patient/symptom-oriented scoring system for oral cGVHD.

### 3.6. Oral Health Quality of Life and Oral cGVHD

Symptoms of oral cGVHD, such as xerostomia, pain and sensitivity, extend beyond physical discomfort, deeply affecting the well-being of individuals. The psychosocial impact is compounded by the persistent and chronic nature of oral cGVHD which may disrupt daily routines and social engagements. In patients with a history of malignancy who have undergone a significant medical event such as alloHCT, distinguishing between generalized and oral health-related anxiety may not be possible. Patients often face a heightened level of anxiety and stress which may be worsened by difficulties with basic activities such as eating, speaking and maintaining social interactions. These challenges can lead to diminished self-esteem, social withdrawal and depression, further exacerbating their overall distress. When patients with oral cGVHD were asked to complete the Oral Health Impact Profile-14, xerostomia and oral sensitivity were found to have the highest negative impact on oral health-related quality of life [123].

## 4. Management of Oral Chronic Graft-Versus-Host Disease

### 4.1. General Considerations

Oral cGVHD management may require a multifaceted clinical approach including systemic immunomodulatory medications, topical and locally applied therapies and supportive care measures [113]. Treatment should be directed to patients with a disruption in the barrier function of the oral cavity, or when oral pain or sensitivity adversely affect the quality of life or oral intake [124,125]. The primary goal of treatment is to reduce symptoms and improve oral health-related quality of life. Patients should be encouraged to maintain routine oral hygiene habits (i.e., brushing 2–3x a day, flossing daily and visiting a dentist regularly). Patients who present with multisystem involvement may initially require systemic treatment, while those who present with cGVHD limited to the oral cavity may be managed with oral-directed therapies only. Patients who are already being managed with systemic therapy may additionally benefit from localized and ancillary measures. Finally, the prognosis and long-term course of oral cGVHD is highly variable and largely unpredictable, without any well-recognized associated factors.

### 4.2. Mucosal Disease

**Figure 4:**
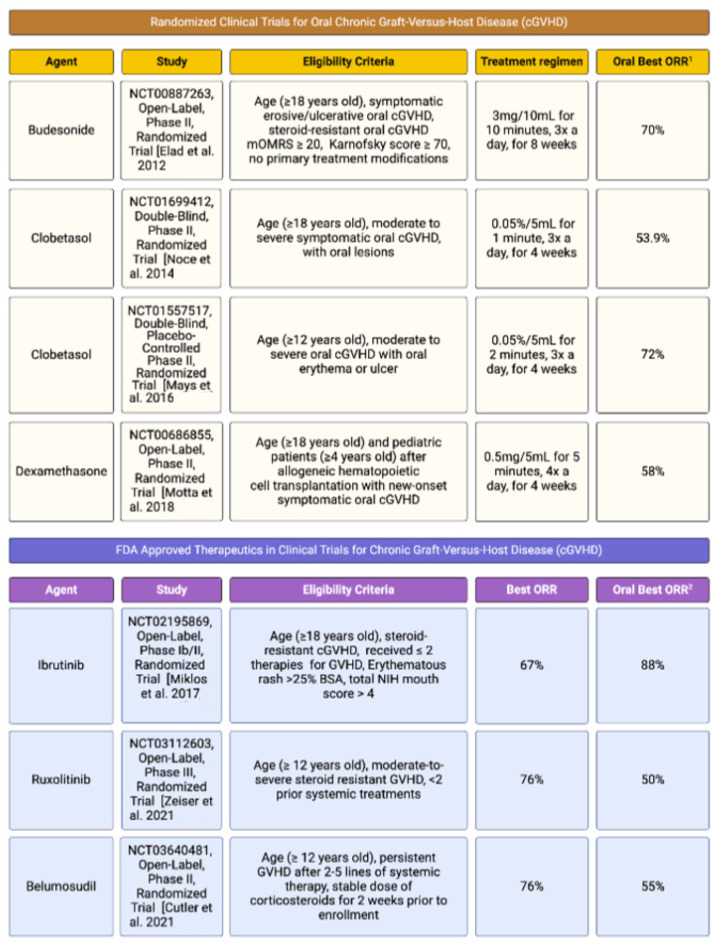
Select chronic GVHD clinical trials reporting oral cavity outcomes. Summary of randomized clinical trials for treatment of oral cGVHD specifically and FDA-approved systemic therapies [145,146,147,148,149,150,151].

Topical therapies, including semi-solids (e.g., gels, creams) and solutions, can play an important role in effective management of oral mucosal cGVHD (Figure 4). At present, there are no FDA-approved topical treatments for oral cGVHD; however, various options are available and are routinely used in clinical practice. When managing widespread oral involvement, the first-line topical steroid therapy is typically dexamethasone 0.5 mg/5 mL solution [126], used as a swish and spit technique to effectively reach all affected surface areas. This is followed by a 15– to 30–min period of no food or fluid intake to ensure maximum contact time. For recalcitrant or severe cases, more highly potent compounded topical solutions (e.g., budesonide 0.03%, clobetasol 0.05%) may be indicated [127,128]. Tacrolimus 0.1% solution alone or in combination with clobetasol 0.05% solution may be considered [127,129]. The use of dexamethasone 0.01%, clobetasol 0.05%, budesonide 0.03% and tacrolimus 0.1% rinses in the management of oral cGVHD are supported by prospective clinical trials [128,129]. When clinicians who regularly manage oral cGVHD were surveyed, 91.7% of the responders used topical steroids as first-line therapy [130].

For limited or isolated areas, or for secondary treatment following use of a solution, gels, creams and ointments (fluocinonide gel 0.05%, clobetasol propionate gel 0.05% and tacrolimus ointment 0.1%) can be applied [127,131]. Application of gels, ointments and creams can be facilitated through a medicament tray (when the lesions are on gingiva) or under gauze occlusion. Due to the concerns for possible malignancy based largely on animal studies, the FDA has issued a “Black Box” warning for tacrolimus ointment. Several studies have evaluated the use of localized phototherapy with [132] psoralen-UVA (PUVA) [133] and narrow-band UVB, but the evidence base is weak and there are potential safety concerns [134]. Adjunctive photobiomodulation therapy has been reported to ameliorate oral cGVHD in case reports and a randomized, double-blind, multi-center trial is ongoing (NCT05675930) [135,136].

Potential complications of the use of topical immunomodulatory agents include oral candidiasis and side effects related to systemic exposure to topical agents [127].

In addition to topical immunomodulatory therapies, some patients may benefit from the use of topical anesthetics and other various topical analgesics and devices intended to provide palliative symptomatic relief of oral mucosal discomfort. These include interventions such as various “magic mouthwash” preparations, which often contain 2% viscous lidocaine, as well as adherent mucosal barrier devices.

### 4.3. Salivary Gland Hypofunction and Dental Considerations

Dry mouth symptoms can be mitigated with frequent water intake and adequate hydration, the use of sugar-free gum and lozenges and over-the-counter dry mouth products such as rinses, gels and saliva substitute [137,138]. Systemic sialogogues including pilocarpine (5 mg, up to three times daily) and cevimeline (30 mg, up to three times daily) may be prescribed to improve salivary flow and associated xerostomia [139]. Optimal oral hygiene maintenance, use of prescription topical fluoride (5000 ppm toothpaste) and regular dental check-ups are crucial to preventing dental caries that can arise due to reduced salivary flow [91].

### 4.4. Sclerodermatous Disease

Physiotherapeutic interventions, including range-of-motion exercises and use of jaw stretching devices, may help to improve symptoms [140]. Severe cases may benefit from intralesional injection of corticosteroids in the fibrotic band or surgical intervention to sever fibrotic banding [113].

### 4.5. Oral Cancer Risk

Patients with oral cGVHD have an increased risk of developing oral squamous cell carcinoma [106]. This risk is believed to be linked to both chronic inflammation and immunosuppression [106,141,142]. Limited data suggest that survival outcomes are comparable to those of non-alloHCT patients [143]. Patients with oral cGVHD should be routinely monitored for mucosal abnormalities and biopsied when indicated [144].

## 5. Emerging Therapies and Future Directions

With advances in the understanding of the etiopathogenesis of cGVHD, there has been progress in the application of more specifically directed therapeutic approaches. Existing therapeutics were repurposed and demonstrated to yield clinical benefit regarding the treatment of oral cGVHD, like budenoside [145], clobetasol [146,147] and dexamethasone [148] (Figure 4). Three targeted molecular therapies have received FDA approval specifically for cGVHD management, including ibrutinib (BTK inhibitor) [149], ruxolitinib (JAK/STAT inhibitor) [150] and belumosudil (ROCK2 inhibitor) [151] (Figure 4).

### 5.1. Ibrutinib

Bruton’s tyrosine kinase (BTK) is a key enzyme in the B-cell receptor signaling pathway, playing a critical role in the development, differentiation and activation of B cells [152,153]. Ibrutinib is a small molecule inhibitor of BTK that was FDA-approved in 2017 for management of cGVHD in adults after failure of one or more lines of systemic therapy [154]. An open-label, multi-center, single-arm clinical trial demonstrated an overall response rate (ORR) of 67% [149,153,155,156] with an oral ORR of 100% [155]. A similar study in Japan demonstrated a best ORR of 73.7% and reported a mouth ORR of 35.7% [156]. Future studies are required to understand the possible benefit of ibrutinib in children.

### 5.2. Ruxolitinib

Ruxolitinib (JAK1/2 inhibitor) blocks the JAK–STAT signaling pathway, which plays a critical role in the activation and proliferation of T cells and the production of pro-inflammatory cytokines [157]. Ruxolitinib received FDA approval in 2019 for the treatment of cGVHD following the failure of one or two lines of systemic therapy in adults [158]. The overall response rate was 70% in the ruxolitinib arm compared to 54% in the best available therapy arm, with a 50% overall response rate in the oral cavity (Figure 4). Topical ruxolitinib is commercially available as 1.5% cream but its effectiveness in treating oral cGVHD is currently being evaluated (NCT03395340) [159]. In children < 18 years old, there was an ORR of 66% with a lower dosing protocol, resulting in a safer and effective outcome; however, the oral cavity was not evaluated [160].

### 5.3. Belumosudil

Rho-associated coiled-coil-containing protein kinase 2 (ROCK2) signaling regulates immune cell activity and the production of inflammatory cytokines [161,162]. Belumosudil received FDA approval in 2021 for treatment of cGVHD in adults and children over the age of 12 after failure of at least two prior lines of systemic therapy [163]. Inhibition of ROCK2 counteracts dysregulation of the adaptive immune system and anomalous tissue repair mechanisms, leading to reduced inflammation and fibrosis [162]. Belumosudil has an ORR of 76%, with a 55% ORR for the oral cavity [163] (Figure 4).

### 5.4. Novel Immunosuppressive Agents

Given the role of pro-inflammatory cytokines such as IL-4, IL-6, IL-17 and TNF-α in the pathogenesis of cGVHD, anti-cytokine therapy can be considered a potential therapeutic approach [164]. Tocilizumab, which inhibits IL-6 receptors [165], has been proposed as a promising treatment option for patients with advanced and steroid-refractory cGVHD [166]. Secukinumab, an IL-17 inhibitor, has demonstrated proof-of-concept efficacy in psoriasiform cGVHD [63]. IL-2 is an inhibitory cytokine that has been studied extensively and has demonstrated efficacy in cGVHD [167,168]. Extracorporeal photopheresis that utilizes ultraviolet-A (UVA) irradiation of the patient’s leukocytes in the presence of 8-methoxypsoralen (a photosensitizing agent) has shown promise in the management of steroid-refractory cGVHD with a meta-analysis-pooled risk ratio of 72% [169].

### 5.5. Future Directions

Oral cGVHD presents a common, complex and multifaceted challenge in post-alloHCT patients, with various clinical manifestations including mucosal inflammation, functional impairment of the salivary glands and tissue fibrosis. The pathogenesis involves a cascade of immunological events starting from conditioning regimens that cause tissue damage and immune dysregulation, leading to a persistent alloreactive state that disrupts tissue homeostasis. The unique aspects of oral cGVHD, such as the interface between the oral epithelium and underlying connective tissue and the involvement of specific immune cells like CD8+ T cells and macrophages, underscore the importance of localized immune responses in disease activity and progression. While systemic corticosteroids and other broadly targeted immunomodulatory therapies form the backbone of cGVHD treatment, the emergence of novel targeted molecular therapies highlights the pivot towards more targeted interventions aimed at specific pathogenic pathways. With further advances in the understanding of oral cGVHD-specific mechanisms, in parallel with advances in pharmacotherapeutics and drug delivery, the future holds great promise. Continued interdisciplinary collaboration and innovation will be crucial to overcoming the challenges posed by oral cGVHD and improving the prognosis for affected individuals.

## Data Availability

No new data were created or analyzed in this study.
